# *IGF2*-derived miR-483 mediated oncofunction by suppressing *DLC-1* and associated with colorectal cancer

**DOI:** 10.18632/oncotarget.10309

**Published:** 2016-06-27

**Authors:** Hengmi Cui, Yuan Liu, Jingrui Jiang, Yangyang Liu, Zhe Yang, Shaogen Wu, Wangsen Cao, Isabelle H. Cui, Chenggong Yu

**Affiliations:** ^1^ Institute of Epigenetics and Epigenomics, Institute of Comparative Medicine and College of Animal Science and Technology, Yangzhou University, Yangzhou, Jiangsu, China; ^2^ Laboratory of Epigenetics & Epigenomics, Medical School, Nanjing University, Nanjing, China; ^3^ Department of Gastroenterology, Nanjing Drum Tower Hospital, Medical School, Nanjing University, Nanjing, China; ^4^ Department of Pathology and Laboratory Medicine, New York Presbyterian-Weill Cornell Medicine, New York, USA; ^5^ Quzhou People's Hospital, Quzhou, Zhengjiang, China; ^6^ Xuzhou Cancer Hospital, Xuzhou, Jiangsu, China; ^7^ Jiangsu Co-innovation Center for Prevention and Control of Important Animal Infectious Diseases and Zoonoses, Yangzhou, China

**Keywords:** IGF2, miR-483, colorectal cancer, DLC-1

## Abstract

Emerging evidence indicates that *IGF2* plays an important role in various human malignancies, including colorectal cancer (CRC). Hsa-miR-483 is located within intron 7 of the *IGF2* locus. However, the mechanism by which increased *IGF2* induces carcinogenesis remains largely elusive. DLC-1 has been identified as a candidate tumor suppressor. In this study, we aimed at investigating whether miR-483 transcription is *IGF2*-dependent, identifying the functional target of miR-483, and evaluating whether tissue and serum miR-483-3p or miR-483-5p levels are associated with CRC. Our results showed that sequences upstream miR-483 had undetectable promoter activity and levels of *IGF2*, miR-483-3p, and miR-483-5p were synchronously increased in CRC tissues. Positive correlations between *IGF2* and miR-483-3p (*r*=0.4984, ***p<0.0001), and between *IGF2* and miR-483-5p (*r*=0.6659, ***p<0.0001) expression were found. In addition, patients with CRC had a significantly higher serum miR-483-5p level (*p<0.05) compared to normal controls. DLC-1 expression was decreased in colorectal cancer tissues and diminished through transient transfection with miR-483-3p. Our results suggest that *IGF2* may exert its oncofunction, at least partly, through its parasitic miR-483 which suppressed DLC-1 in CRC cells. Thus, miR-483 might serve as a new target for therapy and a potential biomarker for the detection of colorectal cancer.

## INTRODUCTION

The incidence and mortality rates of colorectal cancer (CRC) have been increasing rapidly in the past several decades, making it the third most common cancer worldwide [1]. Early detection of CRC appears to be a key measure in reducing cancer related deaths. Fecal occult-blood testing (FOBT), colonoscopy and stool DNA test have been available as screening tests for CRC. However, none of these methods has been well accepted as screening tools due to their low adherence rates, high cost and low sensitivity [1]. An ideal screening test should have a high sensitivity and specificity and should also be safe and affordable so that it can be broadly utilized. *IGF2* (insulin-like growth factor II) is an imprinted gene which is normally expressed exclusively from the paternal allele. An abnormal high expression of *IGF2* has been involved in a variety of cancers, but is expressed at a low level in most normal tissue [2]. We and others have previously reported that biallelic expression of the *IGF2* gene, also known as loss of imprinting (LOI), usually accompanying the overexpression of *IGF2*, occurs in 40% to 50% of tumors, including CRC, and is thought to be an early event in carcinogenesis [3–8]. microRNAs (miRNAs) are 17-24 nucleotide non-coding RNA molecules that regulate a variety of cellular processes including cell differentiation, cell cycle progression and apoptosis [9]. miRNAs have been demonstrated to play an important role in the multistep processes of carcinogenesis either by oncogenic or tumor suppressor function [10]. The study of miRNA has been extended into many kinds of tumors, including CRC. Recent findings have shown that the *IGF2* locus harbors a miRNA, miR-483, within the seventh intron; a positive correlation was found between *IGF2* and miR-483-3p and miR-483-5p expression in the tumors studied [2, 11]. Tumor-associated RNAs have been reported in the serum and/or plasma of cancer patients. Ng *et al.* showed that miR-92 is significantly elevated in the plasma of CRC patients and might be a potential noninvasive molecular marker for CRC [12]. Accordingly, several subsequent studies have shown that miR-483 can serve as potential biomarkers for various cancers [13–15], however the mechanism by which elevated miR-483 impacts the development of cancer remains unclear. The DLC-1 (Deleted in liver cancer 1) gene was originally discovered as a potential tumor suppressor frequently deleted in hepatocellular carcinoma. Its expression is lost or decreased in various cancers including liver, breast, lung, stomach, colon and prostate cancers [16]. Research on DLC-1 has focused on its multiple biological functions in regulating cell skeleton modulation, motion, proliferation and migration [17, 18]. In this study, we evaluated the feasibility of using tissue and serum miR-483-3p/5p as a noninvasive diagnostic test for early detection of CRC and explored the oncofunction of miR-483 and the mechanism of colorectal carcinogenesis through the overexpression of the IGF2 gene and miR-483.

## RESULTS

### Enhanced expression of both miR-483 and *IGF2* in CRC tissues

**Figure 1:**
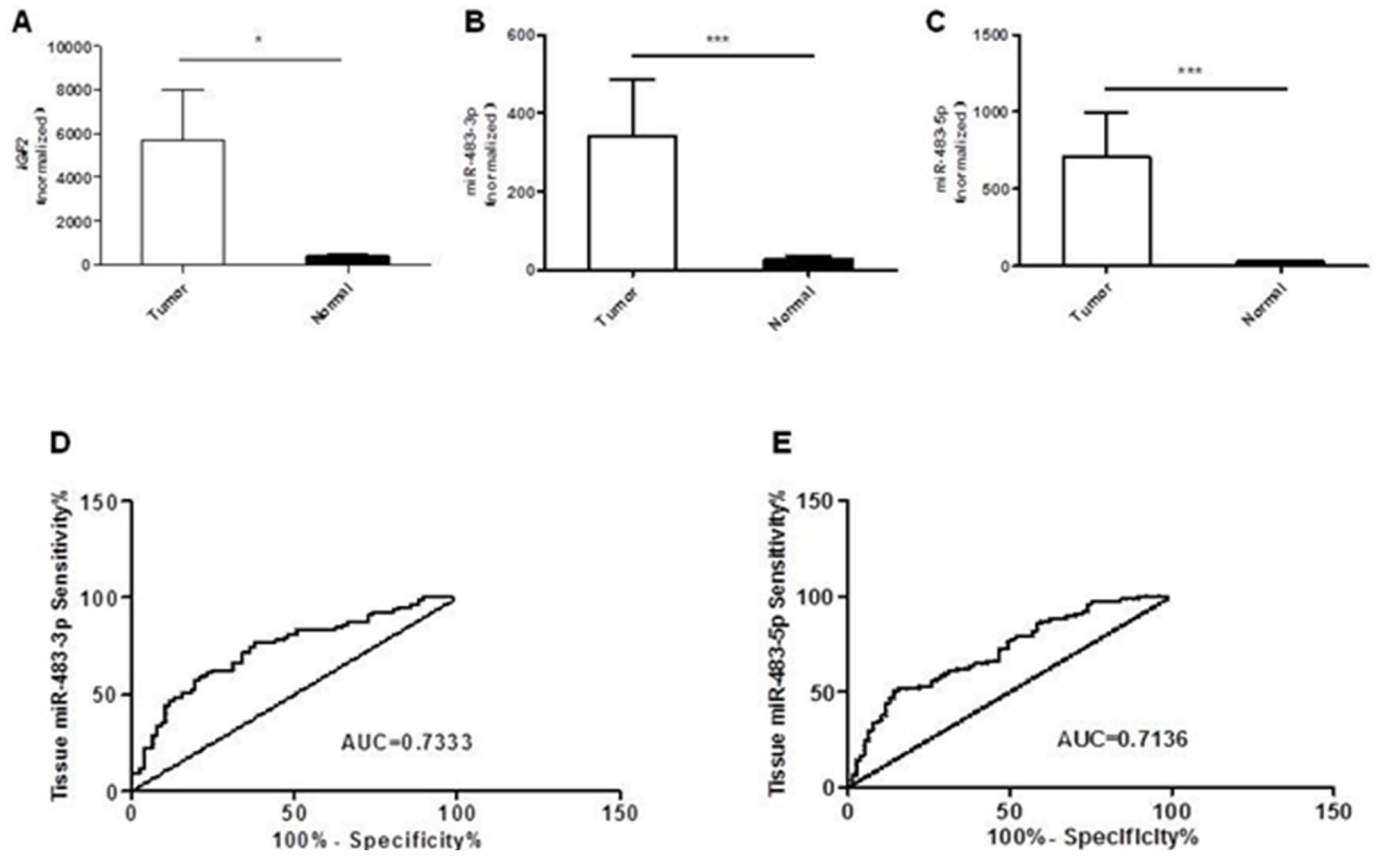
Overexpression of *IGF2*, miR-483-3p and miR-483-5p in colorectal cancers compared to matched normal tissues **A.** Relative expression levels of *IGF2* in colorectal cancer and matched normal tissues (n=77). **B.** Relative miR-483-3p expression levels in colorectal cancer and matched normal tissues (n=77). **C.** Relative miR-483-5p expression levels in colorectal tumor and matched normal tissues (n=77). The expression levels of both miR-483-3p and miR-483-5p were normalized to U6 snRNA and are presented as fold changes (2^−ΔΔCt^) above NC. Receiver operating characteristics (ROC) curves based on **D.** miR-483-3p and **E.** miR-483-5p were plotted to discriminate between normal and CRC patients. MiR-483-3p and miR-483-5p yield an area under the curve (AUC) value of 0.7333 and 0.7136, respectively. *, *p*<0.05 and ***, *p*<0.001 indicate significant or very significant differences in expression levels between paired samples determined by the Wilcoxon matched pairs test.

We examined the expression levels of miR-483-3p, miR-483-5p and *IGF2* in 77 cases of primary colorectal cancers and their adjacent non-cancerous tissues by quantitative RT PCR. When compared to the matched normal tissues, we found that the expression level of *IGF2* was significantly increased in CRC tissues (*p<0.05; Figure 1A). Meanwhile, 59 out of 77 cases showed higher miR-483-3p expression (76.62%, *p*<0.0001; Figure 1B) and 62 had higher miR-483-5P expression (80.52%, *p*<0.0001; Figure 1C). These results suggest that miR-483-3p and miR-483-5p have a similar expression pattern as the IGF2 gene, and all are increased in CRC.

Based on the miRNA levels in CRC and matched normal tissues, the Receiver Operating Characteristic Curve (ROC) had an Area Under the Curve (AUC) value of 0.733 for miR-483-3p (95% CI: 0.6542 to 0.8125; *p*<0.0001; Figure 1D) and 0.714 for miR-483-5p (95% CI: 0.6332 to 0.7940; *p*<0.0001; Figure 1E). A cut-off value, which maximizes the sum of sensitivity and specificity, of 9.22 was selected for miR-483-3p and 12.27 for miR-483-5p. The sensitivity and specificity of miR-483-3p in patients with CRC were 76.62% and 62.34%, and the sensitivity and specificity of miR-483-5p were 51.59% and 84.42%, respectively.

### miR-483 was coexpressed with *IGF2*

**Figure 2:**
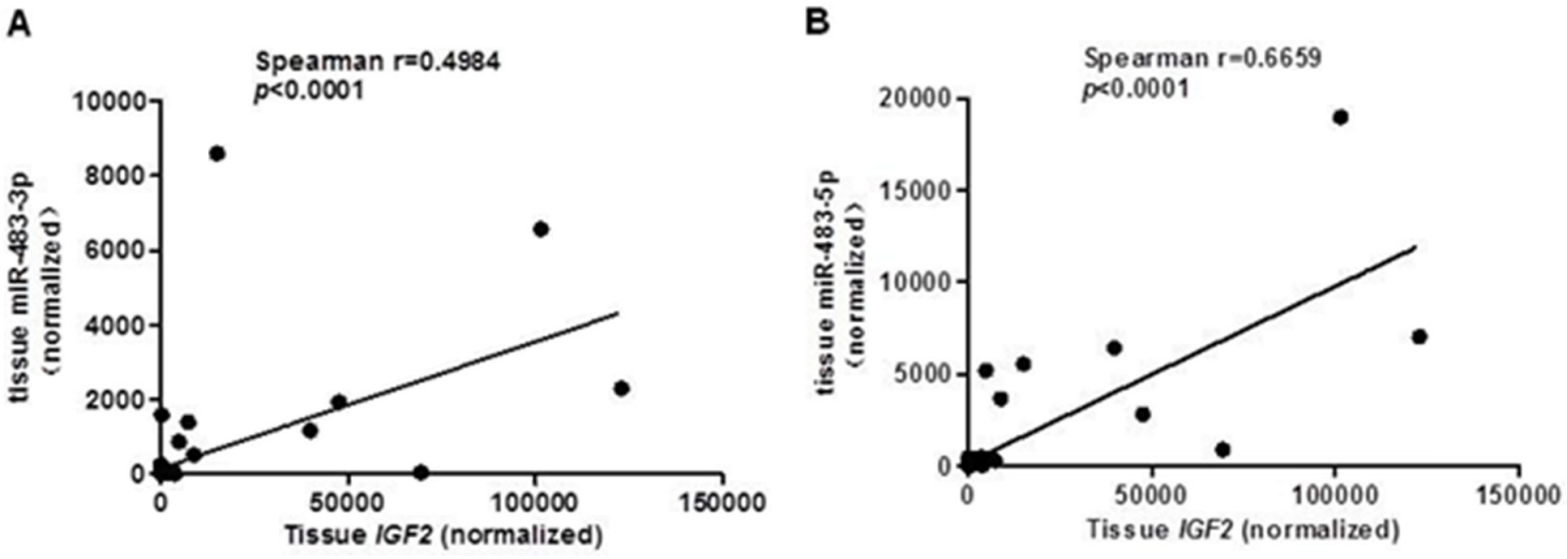
A positive correlation between *IGF2* and miR-483 in CRC tissues The miR-483-3p and miR-483-5p expression levels compared with *IGF2* expression **A.** miR-483-3p expression compared with *IGF2* expression by RT-qPCR (*Rs*= 0.4984, *p*< 0.0001); **B.** miR-483-5p expression compared with *IGF2* expression by RT-qPCR (*Rs*= 0.6659, *p*< 0.0001). The Pearson correlation test was used to determine statistical significance.

**Figure 3:**
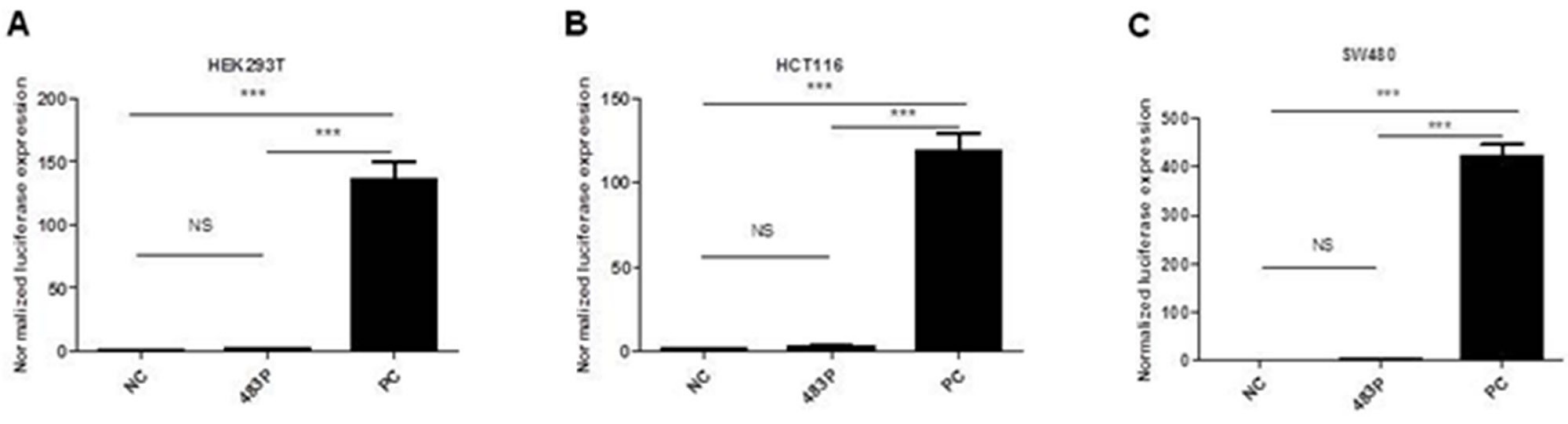
Identification of a potential promoter of miR-483 by luciferase activity assays in HEK293T(A), HCT116(B) and SW480(C) cells No promoter activity was detected within 2kb sequences upstream of miR-483. Cell lines were co-transfected with pGL3-483P containing sequences upstream miR-483(483P); pGL3 basic vector without promoter as a negative control (NC) and pGL3 control vector as a positive control (PC). These vectors were co-transfected with pRL-TK. NS, not statistically significant; ***, *p*<0.001 determined by One-Way ANOVE test.

Since miR-483 is located within the IGF2 gene, we hypothesized that miR-483 may be coexpressed with the IGF2 gene. When compared their expression levels using TagMan RT-qPCR, we found positive correlations between IGF2 mRNA and miR-483-3p (*r*=0.4984, *p*<0.0001; Figure 2A), and between IGF2 mRNA and miR-483-5p expression (*r*=0.6659, *p*< 0.0001; Figure 2B). In order to determine whether miR-483 was co-transcribed with the IGF2 gene, we used the pGL3 Luciferase Reporter vector to detect the putative promoter activity of the 2kb sequences upstream miR-483 and compared that to the luciferase activities of the basic vector or pGL3 control vector; the putative miR-483 promoter activity was undetectable, suggesting that miR-483 may be co-transcribed with the host gene *IGF2* due to a lack of its own promoter (Figure 3A-3C).

### miR-483-5p as a potential CRC marker

In order to select suitable internal controls for serum miRNA detection, the serum expression levels of both U6 snRNA and miR-16 were tested (Supplementary Figures S1A-S1B). We found that only miR-16 expression was consistently detected in all serum samples. No significant difference was detected in the raw Ct values of miR-16 among the normal control (NC) and CRC groups (*p*=0.4050), whereas the U6 snRNA *Ct* value was significantly higher in the CRC group compared to that of the NC (*p*<0.0001). Therefore, miR-16 was selected as the internal normalization control for the serum miRNA detection.

**Figure 4:**
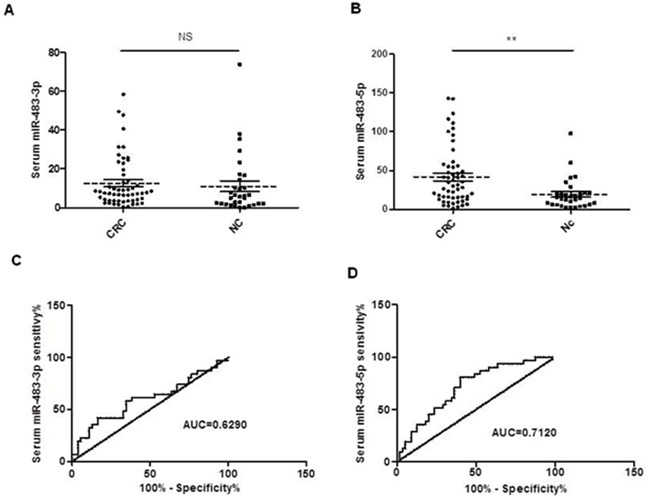
Expression levels of miR-483-3p and miR-483-5p in serum samples from CRC patients (n=55) and normal controls (n=31) The miRNA expression levels were assessed by RT-qPCR and normalized by miR-16. The Mann Whitney test was used to determine statistical significance. **A.** No significant differences in serum miR-483-3p levels were found between CRC patients and normal controls (*p*<0.05). **B.** *A* significant difference was found in serum miR-483-5p level between CRC patients and normal controls (*p*<0.01). Receiver operating characteristics (ROC) curves based on miR-483-3p **C.** and miR-483-5p **D.** were plotted to discriminate normal control from CRC patients. MiR-483-3p and miR-483-5p yield an area under the curve (AUC) value of 0.6012 and 0.7120, respectively. The horizontal solid and dot lines in Figure 1A and 1B represent SEM and mean, respectively.

When comparing serum miR-483-3p and miR-483-5p levels in patients with CRC and healthy controls (CRC, n=55, NC, n=31), we found that serum miR-483-5p level was increased in CRC patients compared to NC (*p<0.01*), but surprisingly there was no significant difference in serum miR-483-3p level between CRC patients and NC (Figure 4A, 4B). ROC curve analysis was utilized to analyze the diagnostic accuracy of serum miR-483-3p and miR-483-5p. The results showed the miR-483-5p AUC for CRC and control was 0.712 (95% CI: 0.6020–0.8220; *p*=0.0012, Figures 4D), whereas the miR-483-3p AUC was 0.6012 (95% CI: 0.4694–0.7329; *p*=0.1219, Figures 4C), indicating that serum miR-483-5p might serve as a potential biomarker for discriminating CRC from normal controls. At the cut-off value of less than 22.15, the sensitivity and the specificity of miR-483-5P were 80.65% and 60%, respectively.

### miR-483-3p suppressed DLC-1 expression and promoted proliferation of CRC cells

**Figure 5:**
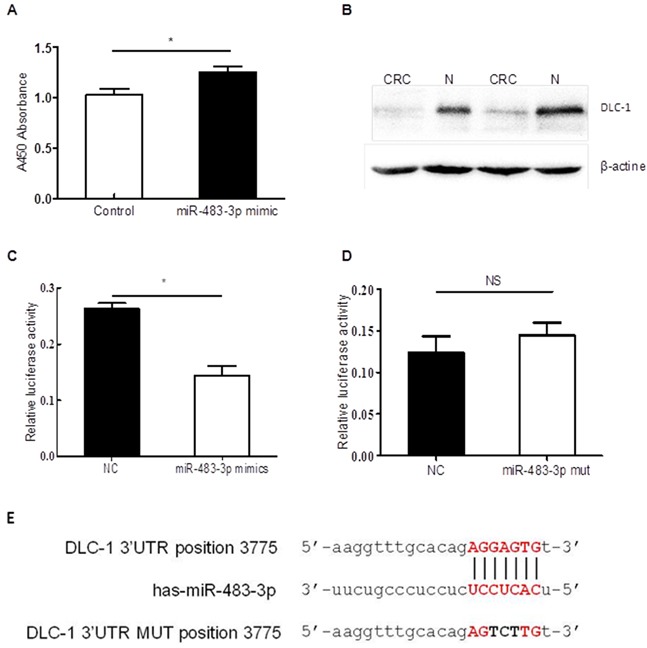
miR-483-3p promoted cell proliferation by specifically targeting *DLC-1 3′*UTR **A.** transfection with miR483-3p mimics promoted cell proliferation. Cell viability was determined at 48h after transient transfection with miR-483-3p mimics or negative control (NC) by CCK8 assay (*p<0.05, non-parametric *t* test); **B.** Western blot analysis showed lower *DLC-1* expression levels in CRC tissues than adjacent normal tissues(N); **C.** Sequence-specific suppression of *DLC-1* expression by miR-483-3p mimics, NC represents negative control; **D.** miR-483-3p mimics could not bind mutant sequences in *DLC-1* 3′UTR. The WT sequences and mutant sequences in DLC-1 3′UTR were cloned into pMIR-GLO vector (pMIR-WT-3′UTR and pMIR-MUT-3′UTR). **E.** miR-483-3p binding sequences in DLC-1 3′UTR are shown on top, miR-483-3p sequences are shown in the middle, and DLC-1 3′ UTR mutant sequences are shown on the bottom. Each of these constructs were transfected into HCT116 cells together with miR-483-3p mimics or negative control sequences and measured after 48h, and normalized using Rluc expression levels as control (*, *p*<0.05, non-parametric *t* test; NS, *p*<0.05).

To study the biological function of miR-483 in colorectal cancer, we used a gain-of-function approach by transfecting the HCT116 cell line with either chemically synthesized miR-483-3p mimics or control oligo sequence. We showed that transient transfection with this miRNA mimic caused changes in cellular proliferation at 48h post transfection using the CCK8 assay (Figures 5A).

After examining the Targetscan and miRbase database, we predicted *DLC-1* to be a putative target gene of miR-483-3p, which may bind to the 3′UTR sequences of the DLC-1 mRNA. We used Western blot to confirm that DLC-1 expression levels were significantly reduced in CRC tissues compared to the matched normal controls. By comparing the DLC-1 protein levels in 10 primary tumor biopsies to its adjacent non-cancerous tissues using Western blot, we found DLC-1 levels in colorectal cancer tissues to be much lower than the adjacent normal tissues (Figure 5B, Supplementary Figure S2, S3).

**Figure 6:**
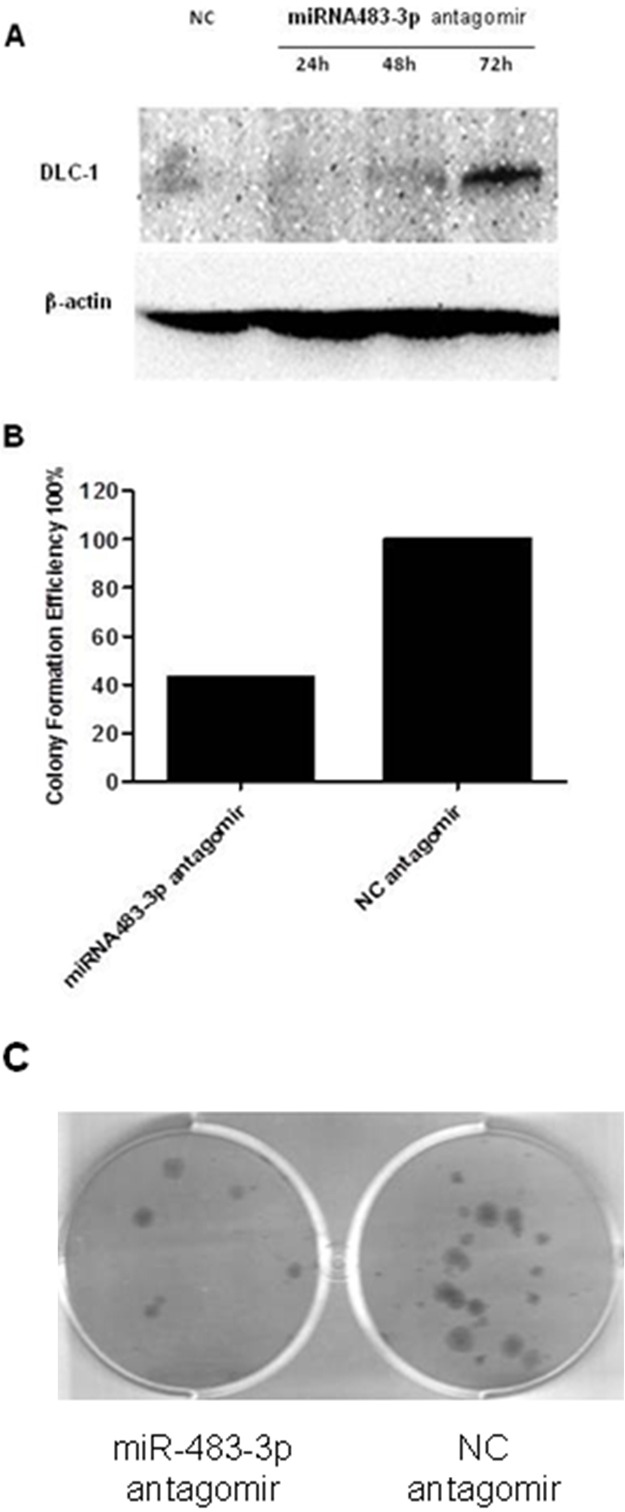
Transfection with miRNA483-3p antagomir induced *DLC-1* expression and inhibited cell growth **A.** Transfection with miRNA483-3p antagomir induced *DLC-1* expression in SW480 Cells. SW480 cells were transfected with either 50nM miRNA483-3p antagomir or negative control antagomir (NC). Immunoblot analysis was performed in SW480 cells transfected with miRNA483-3p antagomir using antibodies against DLC-1 and β-actin; **B.** Inhibition of colony formation in SW480 cancer cells transfected with miR-483-3p antagomir. Cells were stained with crystal violet solution and the number of colonies was counted; **C.** The colony formation on the culture wells

To validate whether *DLC-1* is targeted by miR-483-3p, a dual luciferase reporter assay was performed using constructs in which wild type and mutated sequences were cloned into the reporter vectors (pMIR-WT-3′UTR and pMIR-MUT-3′UTR). When HCT116 cells were co-transfected with targeting vectors pMIR-WT-3′UTR and miR-483-3p mimics, luciferase activities were significantly reduced compared to cells transfected with control sequences (Figure 5C). However, when the seed regions of the targeting site were mutated (pMIR-MUT-3′UTR), the effects of miR-483-3p on luciferase activity were abolished (Figure 5D), suggesting that miR-483-3p may specifically suppress the DLC-1 gene expression, at least in part, by directly binding to its 3′UTR. To further confirm the inhibitory effect of miR-483-3p on *DLC-1* and its oncofunction, a colony formation experiment was performed using SW480 cells which were transfected with either miRNA483-3p antagomir or control antagomir. The results showed that transfection of miRNA483-3p antagomir induce expression of *DLC-1* and inhibit cancer cell growth (Figure 6), further verifying the oncofunction of miR-483-3p.

## DISCUSSION

In this study, we investigated the overexpression of *IGF2* intron-derived miR-483 in tissues and serums of CRC patients and explored the molecular mechanisms by which overexpressed miR-483 predisposes to colorectal cancer. In line with their enhanced expressions in primary tumor specimens, miR-483-5p were also readily detected at high levels in the serum samples of CRC patients, but there was no significant difference in miR-483-3p levels between CRC patient serum and normal control serum. Based on the ROC curves, we selected cut-off values that best discriminated CRC patients from healthy individuals. Tissue miR-483-3p was found to have a sensitivity of 76.62% and a specificity of 62.34%, whereas miR-483-5p had a sensitivity of 51.59% and a specificity of 84.42% for CRC detection. At the cut-off value of less than 22.15 for serum miR-483-5P, the sensitivity and the specificity were 80.65% and 60%, respectively. We showed that miR-483-3p could promote CRC cell proliferation and suppress *DLC-1* expression by directly binding the 3′UTR of mRNA. Finally, we found that transfection of miR-483-3p antagomir could induce the expression of *DLC-1* and inhibit cancer cell growth. It should be noted that the miR-483-3p mimic had less of an effect on cell proliferation than miR-483-3p antagomir. This may be attributed to the HCT116 cells used to testing, which is a malignantly proliferating cell line.

Many studies have shown that intronic miRNAs are coexpressed with their host genes and play a similar role as the host genes [19, 20]. Our results showed that there was undetectable promoter activity in a 2kb fragment upstream miR-483, suggesting that miR-483 may be co-transcribed with the IGF2 gene driven by the *IGF2* promoter. In addition, there was a positive correlation between miR-483 and *IGF2*, both of which were increased in CRC tissues. These results allow us to propose that miR-483 may be coexpressed with its host gene, *IGF2*. *IGF2* has been reported to be involved in the development of many tumors, including Wilms' tumor, hepatocellular carcinoma, breast cancer, bladder cancer, prostate cancer, colorectal cancer, etc. [21–23]. Our group and other labs previously reported that abnormal imprinting or loss of imprinting (LOI) is widely recognized as an important mechanism by which the IGF2 gene promotes carcinogenesis [6–8, 24–29]. This study provides new insight into the mechanism of *IGF2* oncofunction and suggests that the LOI or overexpression of *IGF2* may, at least in part, mediate oncoeffect through the overexpression of miR-483. In other words, the mechanism by which *IGF2* causes carcinogenesis may be at least partially attributed to aberrantly increased miR-483 expression. When this new mechanism was identified to involve in CRC tumorigenesis, we cannot rule out other genetic and/or epigenetic etiologies for CRC due to heterogeneity of cancers.

The first study on miRNAs in colorectal cancer was published in 2002, when miR-143 and miR-145 were identified as novel dysregulated miRNAs [30]. Since then, a lot of other miRNAs have been reported to be involved in carcinogenesis. Tumor-derived miRNAs were first described in plasma by Mitchellet *et al.* in 2008 [31]. The first reported tumor-associated serum miRNA was by Lawrie *et al.,* who found that patients with diffuse large B cell lymphoma had high serum levels of miR-21, which were associated with increased relapse-free survival [32]. miRNAs were then found to exhibit a higher stability over mRNAs in both plasma and serum. Later, miRNAs were also found to exist in a more resilient form which can resist RNase activity [31]. This will allow for a sufficient amount of time for the sample to be transferred to a laboratory for testing, and ensure that subsequent quantitative analysis is less likely to be affected by biomarker degradation. Given that aberrantly expressed blood miRNAs were closely associated with tumor detection, regardless of their derivation, circulating miR-483 would be a good candidate for potential noninvasive biomarker of CRC.

In conclusion, our study evaluated the feasibility of using tissue miR-483-3p, miR-483-5p and serum miR-483-5p as markers for the detection of CRC. We showed that serum miR-483-5p has an acceptable sensitivity for the detection of CRC, making it a potential non-invasive biomarker for CRC screening. Furthermore, by suppressing its target DLC-1 gene and inducing CRC cell proliferation, miR-483-3p may provide a new target for CRC therapy.

## MATERIALS AND METHODS

### Patients

Seventy seven CRC patients with newly diagnosed colorectal cancer and 31 healthy subjects were recruited. Primary tumor biopsies and adjacent non-cancerous colonic tissues (at least 5 cm away from the tumor) from all 77 patients with CRC were collected. Pre-operative serum samples were collected from 55 of these CRC patients. Patients were excluded if they underwent chemotherapy or radiotherapy before blood sampling. Tumors were staged according to the tumor-node-metastasis (TNM) staging system of UICC. Serums from 31 age-matched healthy subjects were collected as the control group based on negative test results which included blood test, abdominal ultrasound examination, rectal exam, fecal occult-blood testing, chest X-ray, CT scan and colonoscopy. None of these controls had previously been diagnosed with any types of malignancy (Supplementary Table S1).

This study was approved by the Review Board of Nanjing Drum Tower Hospital (protocol number 20130086).

### Cell culture and transfection

HEK293T, HCT116, and SW480 cell lines were cultured with Iscove's modified Dulbecco's, McCoy's 5A and RPMI 1640 medium (HyClone, USA), respectively, supplemented with 10% fetal bovine serum (FBS, Invitrogen, USA) and antibiotics (100 IU/ml penicillin and 100 μg/ml streptomycin) at 37°C in a 5% CO2 atmosphere. Chemically synthesized RNAs including miR-483-3p mimics, negative control, and its inhibitor were obtained from Ruibo bioscietech (Ruibo biotechnology, China). Transfection of miRNAs and vectors was performed with Lipofectamine 2000 (Invitrogen) following the procedures of the manufacturer.

### Tissue and serum sample processing and RNA Isolation

All tissue samples were collected during surgery, immediately snap-frozen in liquid nitrogen, and stored at −80°C until RNA extraction. Total RNA was isolated using Trizol (Invitrogen) according to the instructions of the manufacturer.

Peripheral blood was collected in tubes containing separating gel and clot activator and centrifuged at 3500g for 10 min at 4°C and the supernatants were transferred to Eppendorf tubes. A second centrifugation at 12,000g for 10 min at 4°C was performed to completely remove all cellular components. The serum was then aliquoted and stored at −80°C until RNA extraction. All blood samples were processed within 8 h after they were obtained. Total serum RNA was isolated from 400μl serum and eluted in 100μl of RNase-free water using a mirVana™ miRNA isolation kit (Applied Biosystems, shanghai) following the manufacturer's protocol for blood samples.

### RT-qPCR

Individual miRNA tests were performed on independent sets of serum or tissue samples using a two-step procedure. Mature miRNA expression was assayed using the TaqMan MicroRNA assay (Applied Biosystems). For serum miR-483-3p and miR-483-5p detection, miR-16 serum level (PN4427975) was used as the normalized control. First, 10ng of total RNA isolated from 400μl of serum sample or 500ng of total RNA isolated from tissue was subjected to RT using a miRNA specific primer and the TaqMan MicroRNA Reverse Transcription Kit (Applied Biosystems). In brief, RT was conducted in a scaled-down RT reaction volume of 7.5μl, which contained 2.08μl of water, 0.75μl of 10×RT Reverse Transcription buffer, 0.095μl of RNase inhibitor (20U/μl), 0.075μl of dNTPs with dTTP, 0.5μl of Multiscribe™ reverse transcriptase (50 U/μl), 1.5μl of miRNA specific stem-loop RT primer (Applied Biosystems) and RNA preparations. RT was carried out on a Master cycler Epgradient at 16°C for 30 min, 42°C for 30 min and 85°C for 5 min. Thereafter, real-time qPCR was performed using a TaqMan MicroRNA assay (Applied Biosystems) to quantify the individual miRNAs as described previously. The 10μl real-time reaction contained 2.5μl of 1:3 diluted RT product, 5μl of 2X TaqMan^®^ Universal Master Mix II (No AmpErase® UNG, PN4440040), and 0.5μl of 20X TaqMan® assay which contained the forward and reverse primers as well as the TaqMan® probe. Real time PCR was carried out on an ABI PRISM 7300 sequence detection system at 50°C for 2 min, 95°C for 10 min, followed by 40 cycles of 95°C for 15 s and 60°C for 1 min.

Real-time RT-qPCR for mRNA was performed using 500ng of total RNA isolated from tissue according to the instructions of the manufacturer (Takara). Real-time PCR reaction was performed using the TaqMan Gene expression assay instrument. The 10μL PCR reaction included 2μL of 1:3 diluted RT reverse transcription product, 5μl of 2X TaqMan^®^ Gene Expression Master Mix (No AmpErase^®^ UNG, PN4369016), and 0.5μl of 20X TaqMan^®^ assay which contained the forward and reverse primers as well as the TaqMan^®^ probe. The reactions were incubated in a 96-well PCR plate at 95°C for 10 min, followed by 40 cycles of 95°C for 15 s and 60°C for 1 min. TaqMan gene expression assays were performed for IGF2 genes and endogenous reference control gene *GAPDH* using primers and probes (Hs01005963_m1, Hs03929097_g1) obtained from Applied Biosystems.

After completion of the RT-qPCR, the threshold value was manually set above the baseline displayed in the amplification plot. Relative quantification of gene expression was evaluated by utilizing the comparative cycle threshold (Ct). The Ct values for each mature miRNA reaction were subtracted from the respective Ct value of the RNU6B control for tissue samples and the miR-16 control for serum samples to obtain the ΔCt value. The Ct values for each mRNA reaction were subtracted from the respective Ct value of the *GAPDH* control to calculate the ΔCt value. The largest ΔCt value was arbitrarily used as a constant and was subtracted from all the other ΔCt values to determine the ΔΔCt value. Fold changes were then generated for each mRNA and mature miRNA by calculating 2^−ΔΔCT^. All qPCR reactions were performed in triplicate and all Ct values greater than 35 from the real-time PCR assays were treated as 35.

### Luciferase assays and vectors

A fragment of about 2 kb sequences upstream of miR-483, which was assumed to have promoter activity, was amplified by PCR using primers (see Supplementary Table S2) containing the Nhe1 and Xho1 restriction enzyme sites. PCR product was purified, digested, and directly inserted into the pGL3 vector (Promega Corporation, Madison, WI), named pGL3-483P. The pGL3 basic vector was used as negative control and the pGL3 control vector was used as positive control. These vectors with pRL-TK were co-transfected into SW480, HCT116, and 293T cells, respectively.

To generate the pMIR-DLC1-3′UTR construct containing the putative binding site for miR-483-3p within DLC1 3′UTR, human gDNA from 293 cells were used as template and a 660 bp fragment was amplified by PCR using the primers listed in Supplementary Table S2 and was cloned into the pMIR-GLO vector (Promega). We also generated a pMIR-DLC1-3′UTR mutant in which the putative binding site was deleted, named pMIR-GLO-MUT-DLC1. The constructs were confirmed by Sanger sequencing. Each construct was co-transfected into HCT116 cells with 50 nM miR-483 mimics, inhibitor (antisense) or negative control (NC), and 200ng pMIR-GLO-WT-DLC1 or pMIR-GLO-MUT-DLC1 vector. Transfection was performed in OPTI-MEM I (Invitrogen) medium using Lipofectamine 2000 reagent (Invitrogen) according to the manufacturer's protocol. Firefly luciferase activity was measured at 48 hours after transfection using the Dual-Luciferase Reporter Assay System (Promega, USA) which was normalized using Rluc expression level.

### Western blot analysis

Protein was extracted using RIPA buffer (1mM MgCl2, 10 mM Tris–HCl pH 7.5, 1% Triton X-100, 0.1%SDS, 1% NP-40) and the protein samples were either used immediately or stored at −20°C before use. β-actin was used as the loading control. Total protein extracts were separated by 10% SDS–PAGE gels and transferred to PVDF membranes. The level of DLC-1 expression was evaluated using an anti-DLC-1 antibody (1:200, mouse monoclonal anti-human; BD Bioscience). Bands were developed by way of enhanced chemiluminescence reagent and were detected using an auto-exposure system.

### Cell proliferation assays

HCT116 cells were seeded in a 96-well plate at a density of 1×10^4^ cells per well, incubated overnight, and then transfected with either negative control or miR-483-3p mimic through Lipofectamine 2000 (Invitrogen, USA) and cultured at 37°C and 5% CO2 for 48 hours. In vitro growth was measured using the Cell Counting Kit (CCK8) (Dojindo Laboratories, Kumamoto, Japan). The optical density at 450 nm was measured using a Microplate Reader (Bio-Rad, Hercules, CA), and the proliferation index was calculated: experimental OD value/control OD value. The experiment is independently repeated three times.

### Colony formation

HEK293T cells were cultured in DMEM medium supplemented with antibiotics and 10% fetal bovine serum. For colony formation, 4×10^5^ HEK293T cells were transfected using Lipofectamine 2000 (Invitrogen, USA) with either 50nM miRNA-483-3p antagomir or 50nM NC antagomir (RiboBio Co. Ltd., Guangzhou, China) according to the manufacturer's instructions. Twenty four hours later, 50 cells were seeded in 6-well tissue culture plates and continued to culture for 2 weeks. The cells were then stained with crystal violet-formalin solution for 10 min and counted.

### Statistical analysis

The difference in mRNA or miRNA expression levels between paired tissue samples was calculated using the Wilcoxon matched-pairs test. Correlations between independent samplings and RT-qPCR of *IGF2* and miRNA were determined by the Spearman correlation test. The Mann–Whitney test was performed to determine the significance of serum miRNA levels. The area under the curve (AUC) for tissue and serum microRNAs was determined using Receiver Operator Characteristic (ROC) analysis. *P* values <0.05 were considered to be statistically significant. The statistical analysis was performed with software SPSS version 16.0 and graphs were generated using Graphpad Prism 5.0.

## SUPPLEMENTARY FIGURES AND TABLES


