# Supplementary material for: IGF2-derived miR-483 mediated oncofunction by suppressing DLC-1 and associated with colorectal cancer
Source: Oncotarget. 2016 Jun 27;7(30):48456–66. doi: 10.18632/oncotarget.10309 (PMC5217031; doi:10.18632/oncotarget.10309)
Supplement: Supplementary file 1 [file oncotarget-07-48456-s001.pdf]

IGF2-derived miR-483 mediated oncofunction by suppressing *DLC-1* and associated with colorectal cancer

SUPPLEMENTARY FIGURES AND TABLES

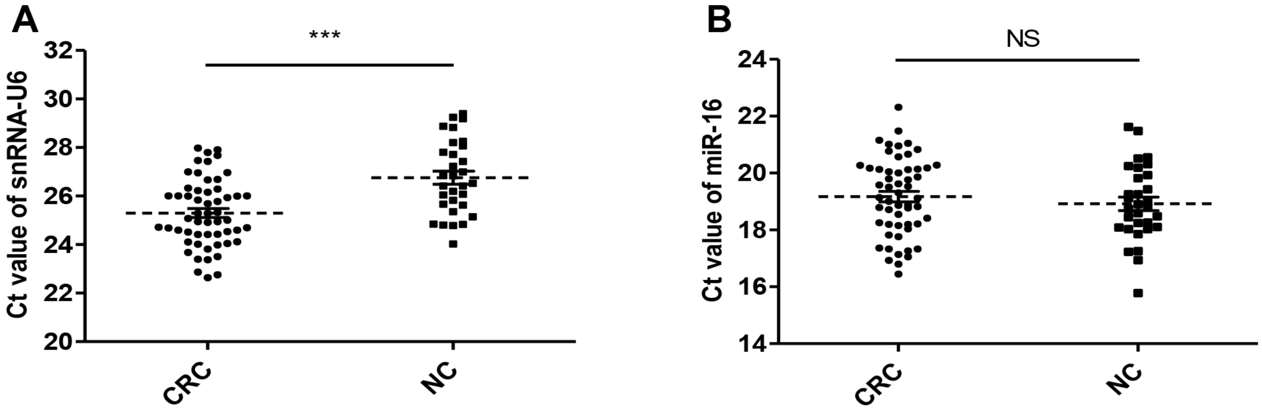

Supplementary Figure S1: Selection of internal control for miR-483 detection by qRT-PCR. A. We compared raw Ct of snRNA-U6 and between serum sample of CRC (n=55) and NC (n=31) groups. CT value of snRNAU6 detected form CRC sample is lower than normal sample. The non-parametrict test was used to determine statistical significance. **B.** We compared raw Ct of miR-16 between serum sample of CRC (n=55) and NC (n=31) groups. No significant differences in raw Ct values of miR-16 were detected among the NC and CRC groups. The non-parametrict test was used to determine statistical significance.

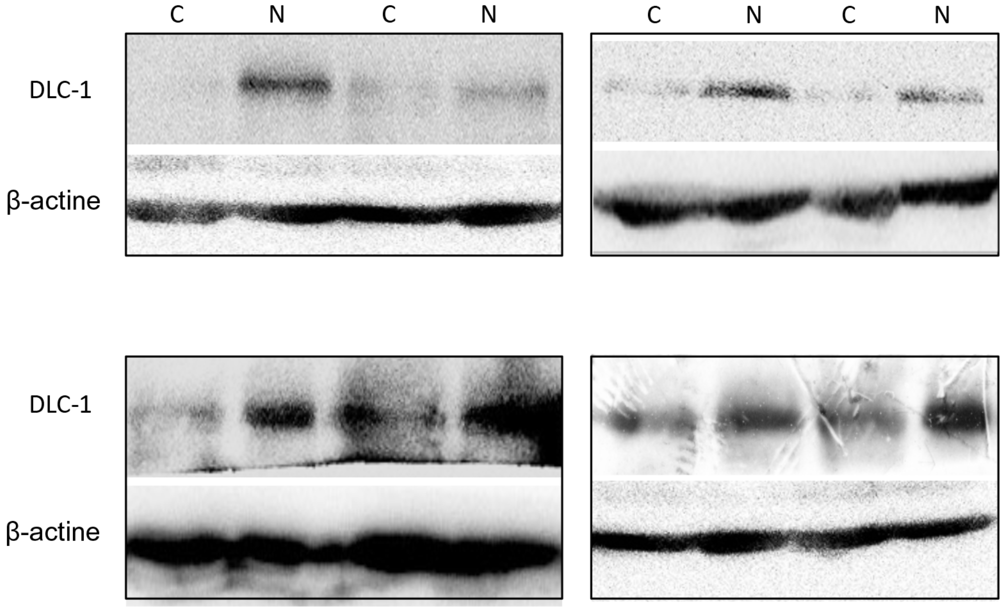

Supplementary Figure S2: Decreased DLC-1 levels in CRC tissues compared to matched normal tissues detected by Western blot. C and N indicate cancer and matched normal tissues.

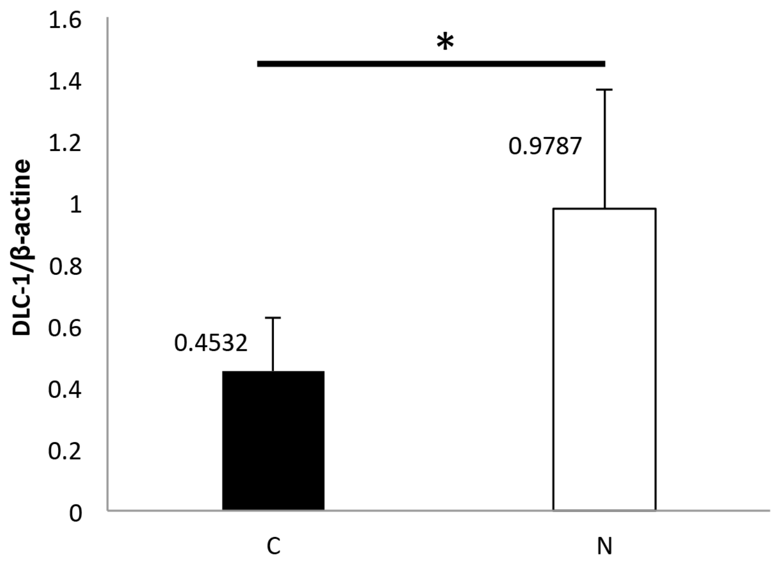

Supplementary Figure S3: Decreased DLC-1 levels in CRC and matched normal tissues (*p*<0.05). The graph was obtained by densitometric quantification of Western blot. C and N indicate cancer and matched normal tissues.

Supplementary Table S1: Clinicopathological characteristics of subjects

	CRC (Tissue)	CRC (Serum)	Normal (Serum)
**No of cases**	77	55	31
**Age at enrolment, years, Mean±SD**	64.78±12.96	64.98±12.98	64.10±9.18
**Gender, No of cases, Men (%)**	40 (51.95%)	31 (56.36%)	18 (58.06%)
**Tumor histology, No of Cases**			
Adenocarcinoma (%)	71 (92.21%)	53 (96.36%)	
Mucinous adenocarcinoma (%)	5 (6.49%)	2 (3.64%)	
Signet ring cell and mucinous (%)	1 (1.30%)		
**Location of tumor, No of cases***			
Proximal (%)	42 (54.55%)	24 (43.64%)	
Distal (%)	35 (45.45%)	31 (56.36%)	
**TNM stage, No of cases**			
I (%)	9 (11.69%)	6(10.91%)	
II (%)	26 (33.77%)	21 (38.18%)	
III (%)	36 (46.75%)	23 (41.82%)	
IV (%)	6 (7.79%)	5 (9.09%)	

* Proximal lesions include tumors at or proximal to the splenic flexure and distal lesions are those distal to splenic flexure.

Supplementary Table S2: Sequences of mature miR-483-3p and miR-483-5p according to miRbase, primers used to amplify ~2000bp fragment of upstream miR-483 and *DLC-1* 3′UTR for promoter activity assay

	Sequences
Mature miRNAs	
has-miR-483-3p	5′-TCACTCCTCTCCTCCCGTCTT-3′
has-miR-483-5p	5′-AAGACGGGAGGAAAGAAGGGAG-3′
Primers for pGL3-483P	
Tag-MiR-483 pmt-F	5′-AGGTACCGCTAGCAAGGTTTCTAGAGCATGGGTGG-3′ KpnI NheI
Tag-MiR-483 pmt-R	5′-ACCCGGGCTCGAGTCCCCCACAGCAATGCTC-3′ SmaI XhoI
Primers for pMIR0GLO-WT-DLC1	5′-CCGCTCGAGGCTTCCTGTTTGTTGAGGGTCT-3′ (F)
	5′-CTAGTCTAGAAAGGCTAGAGGGAGCAGTTCAT-3′ (R)
Primers for pMIR0GLO-MUT-DLC1	5′-TGCACAGAGGCT-GGTGAATGTG-3′ (F)
	5′-ACACATTCACCAGCCTCTGTGC-3′ (R)
